# Immunostimulatory effects of IL-12 targeted pH-responsive nanoparticles in macrophage-enriched 3D immuno-spheroids in vitro model

**DOI:** 10.1007/s13346-025-01896-8

**Published:** 2025-06-16

**Authors:** Maria José Silveira, Cláudia Martins, Ana P. Cardoso, Marc J. K. Ankone, Rhianna R. R. Blyth, Maria José Oliveira, Bruno Sarmento, Jai Prakash

**Affiliations:** 1 i3S – Instituto de Investigação e Inovação em Saúde, Universidade do Porto, Rua Alfredo Allen 208, Porto, 4200-135 Portugal; 2 ICBAS – Instituto de Ciências Biomédicas Abel Salazar, Universidade do Porto, Rua de Jorge Viterbo Ferreira 228, Porto, 4050-313 Portugal; 3 FMUP – Faculdade de Medicina, Universidade do Porto, Alameda Prof. Hernâni Monteiro, Porto, 4200-319 Portugal; 4 IUCS-CESPU, Rua Central de Gandra 1317, Gandra, 4585-116 Portugal; 5 Engineered Therapeutics, Department of Advanced Organ Bioengineering and Therapeutics, Technical Medical Centre, University of Twente, Enschede, 7500AE The Netherlands

**Keywords:** 3D spheroids, Colorectal cancer, IL-12, Immunomodulation, Nanomedicine, pH-responsiveness

## Abstract

Metastatic colorectal cancer (CRC) has the dismal 5-year survival rate of only 14%, and immunotherapies fail to improve the patient outcome. One reason for the poor response rate is the slightly acidic (~ 6.5) immunosuppressive microenvironment. Interleukin 12 (IL-12) is a highly potent pro-inflammatory cytokine that can stimulate tumor immune cells and reverse immunosuppression by inducing interferon gamma (IFN-γ) expression. However, its clinical applications are hindered by systemic side effects. In this study, we developed pH-responsive polymeric nanoparticles (NPs) encapsulating IL-12 to enhance its therapeutic efficacy into the tumor microenvironment (TME). IL-12-loaded pH-responsive NPs induced antitumoral pro-inflammatory response in macrophages at pH ~ 6.5, determined by increased IFN-γ levels and nitric oxide (NO) release, without affecting metabolic activity. In contrast, IL-12-loaded pH non-responsive PLGA NPs showed much lower macrophage activation. To validate the specificity and efficacy in a complex immune-rich microenvironment, we developed a novel CRC 3D immuno-spheroid by incorporating human monocyte-derived macrophages with tumor cells in collagen, mimicking CRC spatial organization and extracellular matrix. The interaction of IL-12 pH-responsive NPs induced macrophage polarization, by providing a reduction of M2-like markers (CD14 + CD163+) while increasing pro-inflammatory M1-like counterparts (CD14 + CD86+). Moreover, IL-12 pH-responsive NPs increased IFN-γ levels and reduced anti-inflammatory IL-10 secretion. Overall, this study provides two major findings (1) a pH-responsive NP system to effectively deliver IL-12 to the TME and reprogram local macrophages into pro-inflammatory phenotype; (2) a macrophage-enriched human 3D immuno-spheroid in vitro system as a tool to test the effectivity of immunomodulatory NPs.

**Supplementary Information:**

The online version contains supplementary material available at 10.1007/s13346-025-01896-8.

## Graphical abstract

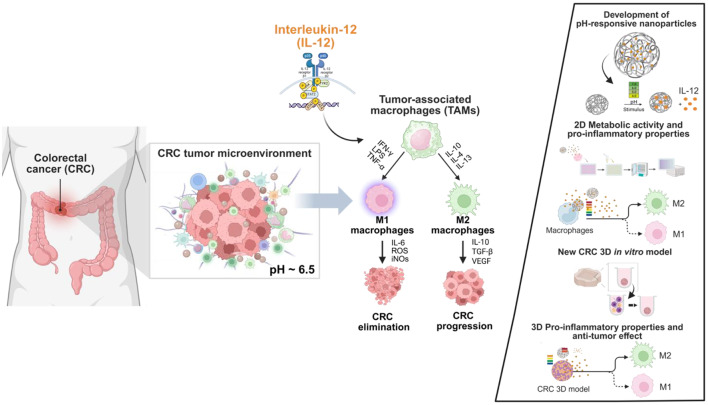

## Introduction

Colorectal cancer (CRC) is the second deadliest cancer worldwide, with up to 50% of localized disease progressing to metastases [1, 2]. Furthermore, metastatic CRC remains a lethal disease with a 5-year survival rate of 14%, underscoring the urgent need of more effective treatments [3].

The tumor microenvironment (TME) plays a key role in regulating metastasis and facilitating mechanisms that promote the invasion of cancer cells [3]. Moreover, in the context of CRC TME, tumor-associated macrophages (TAMs) are being described as one of the most abundant immune cell populations, with a crucial role in the generation of immunosuppressive cytokines that contribute to the promotion of metastasis [4]. In fact, depending on their microenvironment, TAMs can be activated with different features, being categorized as M1-like (promoting an inflammatory response against tumors) or M2-like (supporting tumor advancement through an immunosuppressive phenotype) [5]. Furthermore, recent discoveries have emphasized the importance of activated immune cells in generate and release growth factors and cytokines that modulate the inflammatory microenvironment in CRC [6]. In this context, several immunomodulatory therapies focused on cytokine secretion are being explored for cancer treatment [7].

There are many cytokines which may alter the tumor immune microenvironment such as IL-2, IL-10, IL-22 and IL-12. Cytokines such as IL-10 and IL-22 may act as immunosuppressive or promote cancer cell survival, migration, and proliferation, as well as angiogenesis which can progress the CRC development [8, 9]. Among many cytokines, interleukin 12 (IL-12) has been proposed as a crucial component for immune responses, serving as a key factor in tumor immunotherapy [10, 11]. The primary role of IL-12 involves the differentiation of naïve T cells and activation of macrophages by triggering a signaling cascade of tyrosine kinases JAK and STAT pathways, for IFN-γ cytokine-induced gene expression [12, 13]. Furthermore, IL-12 receptors identified in macrophages contribute to polarization towards M1-like macrophages through IFN-γ induction, fostering an inflammatory microenvironment [14]. Despite its potent antitumor effects in mouse models and initial clinical trials against solid tumors, the benefit-to-toxicity ratio for IL-12 was unfavorable due to IFN-γ -associated toxicity [11]. In addition, the susceptibility of IL-12 to protease-mediated degradation resulted in short half-life and sub-therapeutic concentrations in the bloodstream [15].

Nanomedicine offers a promising solution to improve half-life of protein-based therapeutics and thereby extend their therapeutic effects [15]. TME-responsive drug delivery systems in nanomedicine, activated by stimuli intrinsically present in the TME, have gained enormous interest to achieve tumor-specific drug release [16]. To achieve tumor-specific responsiveness, factors such as size, surface charge, and other physicochemical properties, triggered by TME stimuli such as pH, redox conditions and enzymes, can be modified [17]. Specifically, pH-sensitive nanomaterials have the ability to remain inactive until reaching the acidic TME, enabling pH-triggered, targeted and selective payload release at the tumor site [16]. These nanomaterials act through acid-triggered processes, including functional group protonation and linker cleavage [18]. 2,3-dimethylmaleic amide (DMMA) has been identified as a potent charge conversion molecule and pH-sensitive compound, which undergoes acid-triggered cleavage [19]. Modifying a matrix of poly(lactic-co-glycolic) acid (PLGA), a FDA-approved polymer known for its excellent biodegradable and biocompatible properties, with DMMA might be a promising strategy to further produce pH-responsive nanoparticles (NPs) for localized payload release in the mildly acidic CRC TME (pH ~ 6.5) [19, 20]. This localized action results from the rapid break down of the previously formed PLGA-DMMA amide bond (stable under neutral conditions) upon exposure to the weakly acidic pH of the CRC TME, exposing positively charged amino groups [21].

In this study, we aimed to develop a novel pH-responsive polymeric NPs to deliver IL-12 (termed IL-12 pH-responsive NPs) into the mildly acidic TME and thereby convert pro-tumoral anti-inflammatory macrophages into anti-tumoral proinflammatory macrophages. Furthermore, we aimed to demonstrate their effectivity in human tumor relevant macrophage-enriched 3D immuno-spheroid models. First, we synthesized a PLGA-DMMA conjugate by chemically linking PLGA to DMMA, as confirmed by NMR analysis. The resulting PLGA-DMMA was then used to prepare pH-responsive NPs incorporating IL-12. These IL-12 loaded pH-responsive NPs were characterized for their physicochemical properties, showing rapid release of IL-12 in mildly acidic pH. Moreover, we assessed the effect of IL-12-loaded NPs on the metabolic activity in 2D cell culture models, including the murine RAW 264.7 macrophagic cell line and human primary monocyte-derived macrophages isolated from healthy blood donors. To demonstrate the effectivity of these NPs, we established a macrophage-enriched 3D CRC co-culture immuno-spheroid model embedded in collagen to recapitulate the in vivo immune system, extracellular matrix (ECM) and dynamic cell-cell and cell-ECM interactions. This model demonstrated that IL-12 loaded pH-responsive NPs induced macrophage re-polarization from M2 to M1 type as evidenced by increased expression of the pro-inflammatory marker CD86, elevated secretion of the pro-inflammatory cytokine IFN-γ and nitric oxide (NO), and a concomitant reduction in the expression of the anti-inflammatory marker CD163 and the cytokine IL-10.

## Methods

PLGA 5004 (50:50 LA: GA; 44 kDa; Purasorb^®^ PDLG 5004 A) was kindly provided by Corbion-Purac biomaterials. PLGA-NH_2_ (LA: GA- 50:50; 30 kDa) was purchased from RuixiBiotech^®^ and PLGA-FKR648 was acquired from Akina (USA). Reagents such 2,3-Dimethylmaleic anhydride (DMMA), resazurin, Polyvinyl alcohol (PVA), dichloromethane (DCM), paraformaldehyde (PVA), Sulfanilamide, dimethyl sulfoxide (DMSO), Phosphoric acid, N-naphthylethylenediamine, neutral buffered formalin, trypsin, xylene, Histopaque-1077, a graded alcohol series, hematoxylin and eosin staining solution, Tris, EDTA and sodium citrate were obtained from Sigma-Aldrich (USA). RPMI 1640 and high glucose Dulbecco’s Modified Eagle (DMEM) GlutaMAX medium were purchased from Alfagene, Lda. SeaKem^®^ LE agarose was purchased from Lonza (Switzerland). Accutase^®^ was obtained from Thermo Fisher Scientific (USA), and Triton X-100 from Spi-Chem (USA). The RosetteSep-Human Monocyte Enrichment Cocktail was procured from StemCell Technologies, murine and human IL-12, interferon-γ (IFN-γ), IL-4, IL-13, and bacterial lipopolysaccharide (LPS) were acquired from PeproTech, M-CSF, IL-10 from ImmunoTools (Germany) and Collagen type I (collagen) from Corning (UK). Alexa Fluor 488-anti rabbit, and Alexa Fluor 594-anti mouse antibodies were sourced from Invitrogen (Belgium). Ultrapure water was prepared in-house with a conductivity of 0.055 µS/cm and a resistivity of 18.2 MΩ.cm, using MilliQ^®^ station from Millipore Corporation. For cell cultures, T flasks were acquired from Orange Scientific. Fetal bovine serum and penicillin-streptomycin were acquired from Gibco (USA). IFN-γ and IL-12 ELISA Kit were obtained from BioLegend while IL-10 ELISA KIT from was purchased from PeproTech. The total protein content analysis kit (Dc Protein kit, USA) was acquired from Bio-Rad. Antibodies, including anti-CD14/APC and anti-CD86/FITC were obtained from ImmunoTools. The anti-CD206/PE was purchased from BioLegend. Anti-CD163/PE was acquired from BD Biosciences. The anti-HLA-DR/ PB and anti-EPCAM/PB, Live/Dead Fixable Viability Dye eFluor™ 780 and 4′,6-diamidino-2-phenylindole (DAPI) from Thermo Fisher Scientific^®^. The antibody, anti-collagen type I (Anti-collagen) rabbit was acquired from Rockland Immunochemicals. Dialysis membranes of 10 kDa, 100 kDa, and DMSO-d6 were acquired from Merck (USA). Mouse macrophages RAW 264.7 and the human colon cells SW-48 cell lines were purchased from American Type Culture Collection (Rockville, MD).

### Chemical conjugation and characterization of the DMMA and PLGA polymer composite

The DMMA reagent was conjugated with the PLGA polymer (PLGA-DMMA) in anhydrous conditions, through an amidation process where the carboxylate group present in DMMA reacted with the amide terminal group (–NH2) of PLGA (PLGA-NH_2_) resulting in an amide bond [22]. Briefly, 25 mg of PLGA-NH_2_ (Mn ≈ 30 kDA, 0.83 µmol) was dissolved in 1 mL of DMSO, and 10 equiv (to amino groups) of DMMA (Mn ≈ 126.11 Da,1.05 mg, 8.3 µmol), previously dissolved in 1 mL of DMSO were added. The reaction was stirred for 24 h at 40ºC under inert atmosphere [23, 24].The final solution was transferred into a dialysis bag with 10 kDA molecular weight cutoff and dialyzed for 48 h against 200 mL of distilled water to remove excess DMMA. The composite polymer was then dried under vacuum, in a vacuum oven, overnight at 25ºC. ^1^H NMR measurements were performed with a Varian Inova 400 MHz 1H NMR spectrometer, at room temperature (RT), in deuterated DMSO. Chemical shifts are reported in ppm (δ units) and were referenced to the residual solvent signal. DMSO residual solvent at 2.50 ppm was removed after the analysis using GSD Mnova software (Mestrelab Research) which was used for analysis processing.

### Manufacture of IL-12 loaded pH responsive nanoparticles

NPs containing IL-12 (MW ≈ 75 kDa), were produced using a modified water-in-oil-in-water (w/o/w) double emulsion-solvent evaporation technique [25]. In this process, 20 mg of non-modified PLGA (Mw ≈ 44 kDa, 50/50 ratio of lactide and glycolide, viscosity midpoint 0.4 dl/g viscosity) was mixed with 8 mg of PLGA-DMMA in 4 mL dichloromethane for 3 h at RT. Subsequently, 0.2 mL of 25 ug/mL IL-12 in Phosphate-buffered saline (PBS 1X: 137 mM NaCl, 2.7 mM KCl, 10 mM Na_2_HPO_4_, and 1.8 mM KH_2_PO_4_) was emulsified at 70% amplitude for 30 s using a Vibra-CellTM ultrasonic processor in an ice bath. The initial emulsion was then poured into 4 mL of 2% PVA (aqueous solution) and sonicated for 60 s, resulting in the formation of the second emulsion (w/o/w). The final emulsion was added to 50 mL of PVA. Dichloromethane evaporation from the solution occurred overnight under magnetic stirring at 400 rpm. The NPs were subjected to two rounds of washing with 20 mL of ultrapure water using ultracentrifugation at 8,000 *g* for 30 min at 4ºC.

### Characterization of nanoparticles

#### Average particle size, polydispersity index, surface charge and morphology of nanoparticles

NPs undertook characterization for their Z-average and polydispersity index (PDI) using dynamic light scattering (DLS), while zeta-potential (ζ-potential) was determined through laser Doppler anemometry (LDA) employing a Malvern Zetasizer Nano ZS instrument (Malvern Instruments Ltd). The measurements were conducted on samples diluted in a 10 mM sodium chloride (NaCl) ionic solution, maintaining a particle concentration of 0.2 mg/mL. The reported values represent the mean ± standard deviation (SD) obtained from at least three distinct batches. The morphological analysis of the NPs was performed using scanning Electron Microscopy [26] (JSM-IT100, JEOL, Tokyo, Japan) at an accelerating voltage of 5 kV and a probe current of 38 and 23 respectively.

#### Evaluation of the pH responsiveness of nanoparticles

PLGA-DMMA features a pH-responsive amide linker that remains stable under neutral pH physiological conditions. Upon exposure to a slightly acidic pH environment, the amide linker undergoes rapid hydrolysis, revealing positively charged amino groups. To validate this pH responsiveness of PLGA-DMMA, NPs were subjected to buffer solutions of different pH, namely pH 7.4 and 6.5, and alterations in both their average size and ζ-potential were assessed using DLS/DLA after 24 h of incubation.

#### IL-12 association efficiency and drug loading

The amount of IL-12 encapsulated into NPs was directly quantified by liquid–liquid phase separation. Briefly, NPs were freeze dried after their purification and subsequently solubilized in 4 mL DCM overnight. In the day after, PBS1X in same proportion was added to the first solution to solubilize IL-12. The presence of this cytokine, IL-12, was then measured by enzyme-linked immunosorbent assay (ELISA) as commercially recommended. The association efficiency (AE) was calculated using the following equation [27]:

$$\:AE\:\left(\varvec{\%}\right)=\frac{mass\:of\:encapsulated\:IL-12}{inital\:mass\:of\:IL-12}\:x100$$

### IL-12 in vitro cumulative release

The drug release profile of IL-12 from PLGA-DMMA NPs in an in-vitro setting was assessed through the dialysis diffusion method. The release media comprised potassium phosphate (pH 6.5) and phosphate buffer (pH 7.4). A volume of 100 mL of dissolution media was placed in a flask and maintained at 37 °C ± 2 °C on an orbital shaker. A dialysis membrane with a 100 kDa molecular weight cutoff, containing 1 mL nano-suspension of IL-12, was suspended in the release media. The flask with release media was stirred at 60 ± 2 rpm. At specified time intervals (0.5, 1, 4, 14, and 24 h), 0.2 mL samples were withdrawn, and the amount of drug release was determined using the IL-12 ELISA kit, according to the manufacturer’s instructions. The volume of release media was adjusted to the original volume after each sampling, and the temperature was kept constant. Average percentage release and standard deviations (SD) were calculated (*n* = 3).

### Cell lines and cell culture reagents

The SW48 CRC epithelial cell line was cultured in DMEM supplemented with 10% v/v FBS and 1% v/v penicillin-streptomycin. The cell culture medium was changed every 2–3 days, and trypsin treatment was employed for subculturing the cell lines. The cell cultures were housed in a Cell Culture CO_2_ incubator (ESCO) at 37 °C with 5% CO_2_ and 95% relative humidity. Routine mycoplasma detection was conducted. Additionally, murine macrophages RAW 264.7 were cultured in RPMI1640 medium supplemented with 10% v/v FBS, 100 U/mL penicillin, 0.1 mg/mL streptomycin, and 2 mM L-glutamine in a CO_2_ incubator at 37 °C.

#### Ethics statement

In accordance with the guidelines set forth in the Declaration of Helsinki and with ethical approval granted by the Centro Hospitalar Universitário São João Ethics Committee (protocol reference 90/19), blood donors were duly informed and provided written consent for the utilization of their blood collections for research purposes. Monocytes were isolated from excess buffy coats generously supplied by the Immunohemotherapy Department of Centro Hospitalar Universitário São João (CHUSJ) in Porto, Portugal, sourced from healthy blood donors.

#### Human monocytes isolation

Human monocytes were isolated from healthy blood donors’ buffy coats, as previously reported [28]. In summary, the buffy coats underwent centrifugation and were incubated with the RosetteSep human monocyte enrichment kit (StemCell Technologies) to isolate peripheral blood mononuclear cells (PBMCs). Subsequently, each sample was diluted 1:1 with PBS 1X supplemented with 2% FBS and layered over Histopaque-1077 (Sigma-Aldrich) to separate monocytes from other blood components. The enriched monocyte layer was collected and washed with PBS 1X.

#### 2D macrophages differentiation

Murine RAW264.7 macrophages were differentiated towards an M1-like phenotype with murine recombinant 20 ng/mL IFN-γ and 100 ng/mL LPS. Alternatively, RAW264.7 macrophages were differentiated towards an M2-like phenotype using 10 ng/mL murine recombinant IL-4 and 10 ng/mL IL-13. For the differentiation of monocytes isolated from blood donors into macrophages, 0.2 × 10^6^ monocytes (in 24-well plates) were cultured and differentiated using 50 ng/mL M-CSF for a period of 7 days, in RPMI1640 medium over glass coverslips. Following a 7-day differentiation period, the medium was aspirated, and the macrophages were subjected to a washing step. To serve as controls for polarization, human macrophages were exposed to 10 ng/mL LPs (M1-like) or 10 ng/mL IL-10 (M2-like). M0 macrophages, on the other hand, were cultured without the addition of any exogenous factors. The cells were maintained in a humidified atmosphere at 37 °C and 5% CO2.

#### Assessment of the metabolic activity in 2D macrophages cells

Resazurin assay was used to analyze cell metabolic activity. For murine RAW264.7 macrophages, 0.15 × 10^6^ cells were seeded in 96-well plates for 24 h and for human monocytes 0.2 × 10^6^ cells, were differentiated in 24 well plates for 7 days, as described above. Subsequently, after 24 h and 7 days, respectively, the medium was removed, cells were washed with PBS 1X, and different concentrations of free IL-12 or NPs (62,5, 125, 250, 500 pg/mL regarding drug content) in medium (200 µL) were incubated for 24 h, at 37 °C. Afterwards, resazurin redox dye (0.01 mg/mL) was added (1/10 of the total volume of culture medium) to cell culture, incubated for 4 h at 37 °C and 5% CO2, and the fluorescence intensity was measured at 530/590 nm using a SynergyMx MultiMode microplate reader (BioTek). All data were normalized using a background control and positive control (M0 cells without treatment, 100% metabolic activity). The cell metabolic activity was analyzed using as a threshold of 70% cell viability according to the ISO 10993-5 standard [29]. The pH responsiveness of the NPs was confirmed using the phenol red indicator.

#### Nitric oxide (NO) release bioassay in 2D macrophages

The impact of the developed NPs on inducing M1 proinflammatory polarization in both murine and human macrophages was evaluated by assessing NO release, a stable metabolite produced by M1-like macrophages. Macrophages isolated from healthy blood donors were cultured as mentioned above. RAW264.7 cells (0.15 × 10^6^ cells/well) were seeded in 96-well plates and cultured for 24 h. Subsequently, the medium was replaced, and varying concentrations of free IL-12 or different NPs (62.5, 125, 250, 500 pg/mL with respect to the IL-12) in medium (200 µL) were incubated for an additional 24 h at 37 °C. Controls for M1 polarization were performed using IFN-γ (20 ng/mL) and LPS (100 ng/mL), while M2 polarization controls involved incubation with 10 ng/mL IL-4 and IL-13. For macrophages isolated from healthy blood donors, following a 7-day differentiation period, both free IL-12 and NPs in the same concentrations as aforementioned were added. In parallel, controls for polarization were conducted using 10 ng/mL LPs (M1-like) or 10 ng/mL IL-10 (M2-like). In both cases, M0 macrophages were cultured without the addition of any exogenous factors. After 24 h, 100 µL of the culture supernatant was combined with 100 µL of Griess reagent (1% sulfanilamide, 0.1% naphthyl ethylenediamine dihydrochloride, 3% phosphoric acid), and the absorbance at 540 nm was measured using a microplate reader. NO release assays were conducted in triplicate across three independent experiments. The pH responsiveness was confirmed using the phenol red indicator.

#### In vitro efficacy studies in 2D macrophages by IFN-γ production

To initiate the differentiation of RAW macrophages, cells were seeded at a density of 0.15 × 10^6^ cells per well in 96-well plates and incubated for 24 h. Following this incubation, the medium was replaced, and the IL-12 pH-responsive nanoparticles (NPs), free IL-12, and respective controls were added at concentrations of 62.5, 125, 250, and 500 pg/mL (with respect to the IL-12) for a 24 h incubation period. M1 or M2 polarization controls were implemented by exposing the cells to LPS (100 ng/mL) and IFN-γ (20 ng/mL) for M1 differentiation, and IL-4 (10 ng/mL) and IL-13 (10 ng/mL) for M2 differentiation, also for 24 h. In the case of human macrophages, after a 7-day differentiation period, both free IL-12 and NPs at the aforementioned concentrations were introduced. Furthermore, polarization controls were conducted using either 10 ng/mL of LPs (M1-like) or 10 ng/mL of IL-10 (M2-like). M0 macrophages in both scenarios were cultured without the addition of any exogenous factors. Subsequently, IFN-γ concentration in the supernatants was determined by ELISA using a commercially available kit according to the manufacturer’s instructions. All efficacy studies were conducted independently at least three times. The pH responsiveness was confirmed using the phenol red indicator.

#### 3D colorectal cancer immuno-spheroid establishment

Multicellular colorectal spheroid, comprising SW48 cells, human monocyte-derived macrophages, and collagen, were generated using commercially available molds (3D Petri Dish^®^, MicroTissues, USA). Agarose was dissolved at a concentration of 2% (w/v) in 0.9% NaCl (w/v) and poured into 3D Petri Dish^®^ molds to create hydrogel micro-molds featuring 81 uniform circular recesses. Each micro-mold was filled with 190µL of medium and placed in a well of a 12-well plate containing 2 mL of medium [30, 31].For monoculture, SW48 cells (0.0025 × 10^6^, 0.005 × 10^6^, or 0.01 × 10^6^ cells per spheroid) were added to the micro-molds (190 µL) and allowed to settle for 30 min before supplementing each well with complete DMEM medium (2 mL). In the case of dual culture control spheroid (SW48 and human monocytes), the total number of cells per spheroid was maintained at 0.005 × 10^6^. Additionally, three different ratios were explored for human monocytes: SW48 cancer cells double culture (75:25, 65:35, and 50:50), and a defined concentration of collagen (8 µg/mL) was incorporated to mimic the presence of the extracellular matrix (ECM). Macrophage differentiation within the spheroids, originated from human peripheral monocytes, occurred spontaneously in situ without the need for colony-stimulating factors or other exogenous agents, as previously reported by our group [31]. The medium was refreshed every two days.

#### Characterization of the average diameter, metabolic activity, and circularity of 3D colorectal spheroids

3D spheroids images were captured at specified time intervals (1, 4, and 7 days) using brightfield microscopy (ZOE™ Fluorescent Cell Imager, Bio-Rad Laboratories, USA). The 3D spheroids’ average diameter, circularity, and compactness were randomly assessed in three distinct spheroids per micro-mold core through the AnaSP open-source software [32]. For metabolic activity measurements on days 1, 4, and 7, 3D spheroids were incubated with DMEM complete medium containing resazurin (20% v/v) and incubated in the dark at 37 °C for 4 h. Following incubation, the fluorescence of was measured at the excitation and emission wavelengths of 530 nm and 590 nm, respectively, using a SynergyMx™ MultiMode microplate reader (BioTek™, USA). All analysis were conducted on samples from three independent blood donors, with each analysis performed in triplicate.

#### Histological analysis of the 3D colorectal spheroids

On day 7 of culture, the medium was extracted from micro-molds, and the 3D spheroids were fixed in neutral buffered formalin (10% v/v) for 24 h at RT. Subsequently, the micro-molds underwent a PBS 1X wash, and a sealing layer of 2% w/v agarose was applied to the top of each micro-mold. The micro-molds were then embedded in paraffin using an automated embedding system (Thermo Scientific™ STP 120 Spin Tissue Processor, USA). Paraffin-embedded samples were sectioned into 3 μm sections, deparaffinized in xylene, and rehydrated in a graded alcohol series. Staining was carried out using hematoxylin and eosin (H&E), Sirius Red (SR), and Masson’s Trichrome (MT).

#### 3D colorectal spheroids Immunofluorescence analysis

At day 7, the fixed paraffin-embedded samples, prepared as outlined earlier, underwent sectioning into 5 μm sections, followed by deparaffinization in xylene and rehydration in sequentially decreasing ethanol concentrations. Prior to staining, sections were subjected to antigen retrieval by treatment with Tris-EDTA (TE) (pH 9) or sodium citrate buffer (pH 6) at 96 °C for 30 min. Subsequently, the samples underwent three washes by immersing sections in PBS 1X (pH 7.4) for 5 min each [30]. For sample permeabilization, sections were immersed in 0.25% (v/v) Triton for 10 min and then washed three times, as previously described. Before the addition of antibodies, samples were blocked with 10% FBS in PBS 1X for 1 h at RT. Primary human antibodies diluted in block solution (Anti-EpCAM 1:100, Anti-CD68 1:100, and Anti-collagen 1:100) were introduced to the samples and incubated overnight in a wet chamber at 4 °C. Following three washes, secondary antibodies (at a 1:400 dilution) diluted in block solution were incubated in a wet chamber, in the dark, for 1 h at RT. Subsequently, samples were stained with DAPI (1:25,000) solution for 20 min and after washed for 5 min in PBS 1X. Finally, sections were mounted with VectaShield and imaged using a Zeiss AxioImager Z1 microscope (Carl Zeiss, Germany) equipped with an AxioCam MR ver. 3.0.

#### Characterization 3D colorectal spheroids through flow cytometry analysis

Following a 7-day culturing period, 3D colorectal spheroids were gathered into a 15 mL Falcon tube, centrifuged (300 g, 5 min, 20 °C), and the medium was collected. The samples were then washed once with PBS 1X through centrifugation under the same conditions, followed by the removal of the supernatant. Dissociation of the 3D colorectal spheroids into a single-cell suspension was achieved by incubating with Accutase for 15 min at 37 °C, with periodic pipetting every 5 min during the incubation. Subsequently, cold PBS 1 × (1.5 mL) was added to inactivate the Accutase, and the cells were centrifuged (300 g, 5 min, 4 °C) and washed with PBS 1X. Next, 200,000 cells per staining were resuspended in FACS Buffer containing PBS 1X, 2% BSA, and 0.01% azide and centrifuged again. Following that, conjugated antibodies (anti-EpCAM/PB, anti-CD14/APC, anti-CD86/FITC, anti-CD163/PE, anti-CD206/PE, and anti-HLA/DR/PB), previously diluted 1:50 in FACS buffer, were incubated for 40 min at 4 °C in the dark. After incubation, cells were washed twice with FACS buffer and incubated with Live Dead (1:10,000) diluted in PBS 1X for 20 min. Following washing with FACS buffer, samples were incubated with 2% PFA for 15 min at RT. Finally, samples were washed again in FACS buffer and filtered through a 70 μm pore filter membrane. Cell counting beads were used to absolute cell count of each sample in the concentration of 9.7 × 10^− 4^ particles/mL. The acquisition of samples was performed using the BD FACS Canto™ II flow cytometer (BD Biosciences, USA), and all analysis were performed with FlowJo software (Tree Star, Inc., USA). Cells submitted to the same protocol with only FACS buffer were the unstained control. Experiments were conducted at least in triplicate.

#### Uptake of NPs into 3D colorectal cancer spheroids

On the 6th day of 3D spheroid development, empty FKR648-labeled NPs in DMEM without FBS (100 µg/mL) were introduced to each micro-mold and incubated for 24 h. Following incubation, the medium was collected, and the 3D spheroids were washed with PBS 1X. Subsequently, the spheroids were transferred to a 15 mL plastic tube. Within each tube, spheroids were subjected to trypsin incubation for 15 min at 37 °C, followed by vigorous pipetting for dissociation into single cells. The isolated cells were then washed twice with PBS 1X, fixed with 2% PFA for 20 min at room temperature (RT), washed again, and placed into cytometer tubes for subsequent analysis. BD FACS Canto™ II flow cytometer was employed for cytometry acquisition. Cells incubated solely with cell culture medium served as the unstained control, and all cell constituents were grouped into a single gate. All data was processed using FlowJo software. Experiments were conducted at least in triplicate.

#### Effect of NPs on macrophages polarization and anti-proliferative effect of 3D colorectal cancer spheroids

At day 6, each micro-mold was washed with PBS 1X, and different concentrations of IL-12 or NPs (62.5, 125, 250, and 500 pg/mL, in regard to the IL-12) in medium without FBS (190 µL per micro-mold, and 2 mL per each 12 well-plate well where the micro-mold was placed) were added and incubated for 24 h, at 37 °C. Afterwards, the flow cytometry staining protocol was applied as described above. The cells acquisition was performed in BD FACS Canto™ II flow cytometer and analyzed with the FlowJo software. The pH responsiveness was confirmed using the phenol red indicator.

#### Effect of NPs on NO, IFN-γ and IL-10 levels of 3D colorectal cancer spheroids

Following the incubation of nanoparticles (NPs) and their respective controls, 3D colorectal spheroids were evaluated for nitric oxide (NO) release and their capacity to generate pro- or anti-inflammatory cytokines (IFN-γ or IL-10). The evaluation of NO release was conducted as previously described, while cytokine production was determined using ELISA Kits from the selected manufacturers. The pH responsiveness was confirmed using the phenol red indicator.

### Statistical analysis

The findings were presented as mean ± standard deviations (SD) derived from a minimum of three independent experiments. Statistical analysis involved two-sided unpaired Student’s t-test for comparing two independent groups and one- or two-way ANOVA for comparing three or more groups, conducted using GraphPad Prism 8.0 software (GraphPad, USA). Significance levels were established at probabilities of *p < 0.05, **p < 0.01, and ****p < 0.001.

## Results and discussion

### Synthesis and characterization of the pH-responsive NPs

**Fig. 1:**
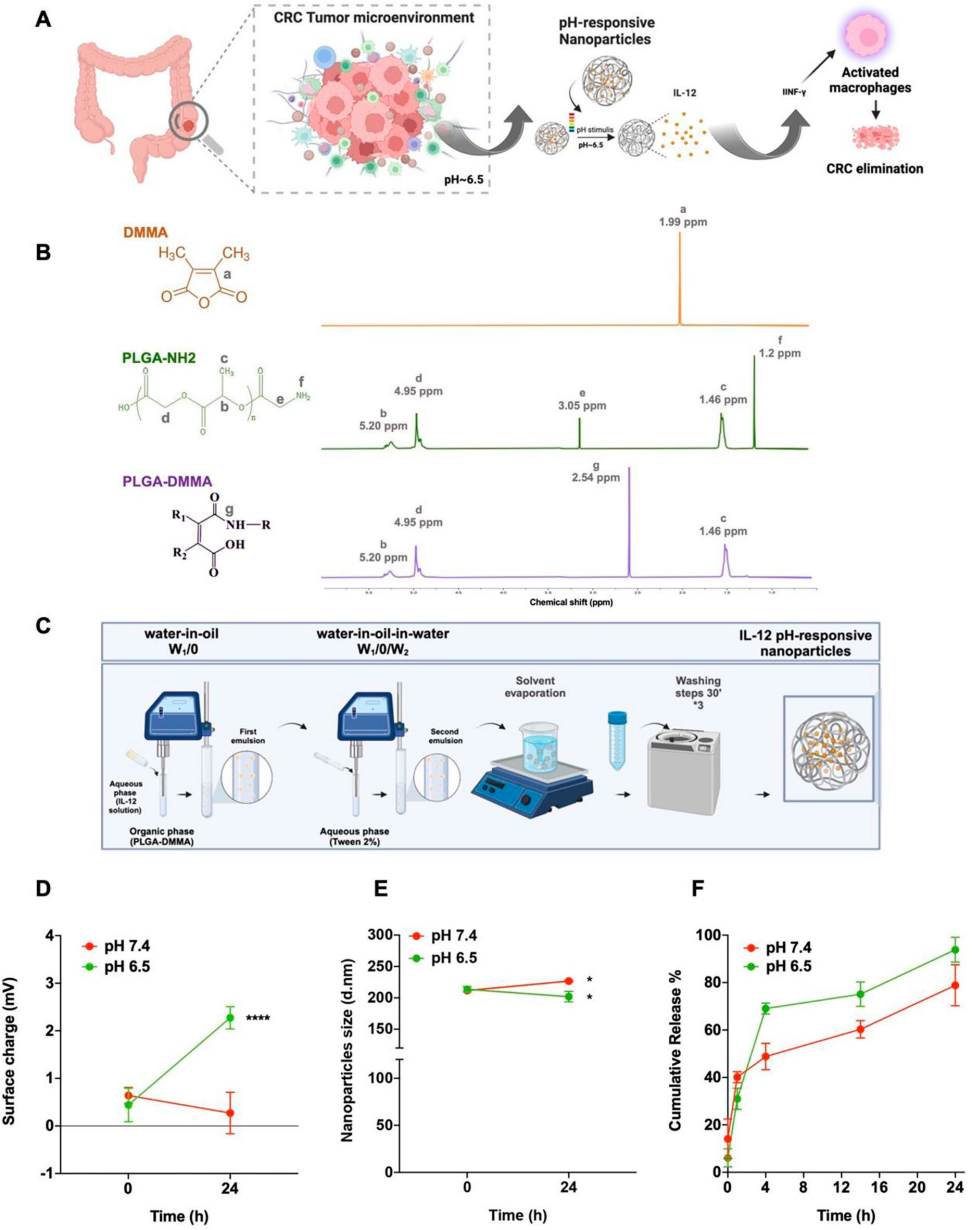
Schematic Illustration of the primary objectives of the developed strategy and its physicochemical characterizations. **A**) Illustration outlining the intended objectives of NPs. **B**) 1 H NMR characterization of PLGA-DMMA polymer performed in DMSO-d6: characteristic peaks of glycolic acid (δ = 5.21, 1.47 ppm), lactic acid (δ = 4.89 ppm), and DMMA (δ = 2.54 ppm) in the final PLGA- DMMA polymer. **C**) Demonstration of the double emulsion technique used in the manufacturing of NPs. **D** and **E**) Assessment of the surface charge and size of NPs submitted at pH 7.4 and 6.5, respectively. **F**) In vitro release of IL-12 from pH- responsive NPs at pH 7.4 and 6.5 at 37ºC over 24 h. All measurements were conducted in triplicate, and results are presented as mean +/- SD. Statistical comparisons made using two-way or one-way ANOVA, *p < 0.05, **p < 0.01, ***p < 0.001 or ****p < 0.0001

To deliver IL-12 efficiently into the slightly acidic CRC TME, pH-responsive NPs encapsulating IL-12 were prepared and characterized (Fig. 1A). To chemically obtain the pH-responsive NP matrix, a PLGA core polymer (30 kDa, LA: GA-50:50) was selected due to its moderate degradation rate and ability to provide controlled drug release, and the DMMA moiety was chosen due to its pH-sensitive cleavable properties. An amide bond was formed between PLGA and DMMA, expected to be stable at physiological pH (pH = 7.4), but cleavable in the mildly acidic conditions of the CRC TME (pH = 6.5), as shown earlier [30, 31]. ^1^H NMR data confirmed the successful reaction between the carboxylate group of the DMMA and the amine terminal group of PLGA, revealing characteristics peaks of PLGA (δ = 5.20, 4.95 and 1.46 ppm [25]; NH_2_ specifically at δ = 3.05 and 1.2 ppm [33]) and DMMA (δ = 1.99 ppm [34]) (Fig. 1B). The appearance of a distinct peak at δ = 2.54 ppm in the spectrum of the final polymeric conjugate is associated with the C = O stretching vibration of the carboxylic acid on DMMA [33]. This observation strongly suggests the successful integration of DMMA into PLGA (Fig. 1B).

Following the polymer conjugation, the newly synthesized PLGA-DMMA was used to produce IL-12-loaded pH-responsive NPs by double emulsion technique (Fig. 1C). IL-12 was encapsulated with an association efficacy of 46.8 $\:\pm\:\:$5.4% (Table 1). This finding aligns with the IL-12 solubility profile and molecular weight with high aqueous solubility, possibly indicating part of its diffusion into the aqueous external phase during the encapsulation process [15, 35].

The physicochemical characteristics of NPs revealed a Z-average size of around 200–250 nm, with a uniform particle size distribution, indicated by a low polydispersity index of only around 0.1. The ζ-Potential approached a near-neutral surface charge, attributed to possible adsorption of negatively charged IL-12 molecules to -NH2 groups from PLGA in the NP structure [36, 37].

**Table 1:**
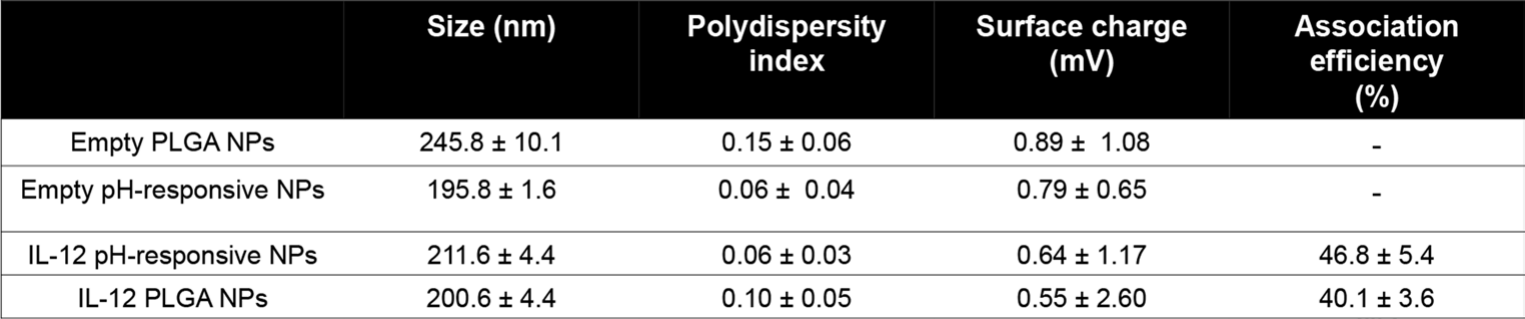
Physicochemical characteristics of empty pH-responsive NPs, IL-12-loaded pH-responsive NPs, and their respective controls were assessed, including mean size (Z-average size), polydispersity index, surface charge (ζ-Potential), and association efficacy (AE %). Values are presented as mean ± SD (*N* = 8). Non-applicable values are denoted by (-)

We then evaluated the effect of pH on the surface charge and size of the developed NPs (Fig. 1D-F). Significant differences in the ζ-Potential of NPs were observed after 24 h of incubation at pH 6.5 (Fig. 1D), which is in line of the previous reported studies using DMMA as a monomer capable of inducing cationic charge reversal properties under slightly acidic pH conditions [21, 38, 39]. Moreover, the modification of NPs with the pH-responsive monomer DMMA has been described to induce average size alterations at slightly acidic pH conditions [40]. Thus, following a 24 h incubation at different pH values, significant variations in the average size of NPs was found at pH 6.5, with a decrease in the average size being associated with the degradation of DMMA (Fig. 1E) [40].

Furthermore, to demonstrate the pH responsiveness of the NPs, the cumulative release of the encapsulated IL-12 was assessed at different pH buffers (Fig. 1F). Notably, both at pH 6.5 and 7.4, there was an initial burst release of about 40% IL-12 at t = 1 h which is likely due to release of IL-12 from the outer layer of NPs. Importantly, there was a rapid release of IL-12 (~ 70%) at pH 6.5 compared to pH 7.4 (~ 45%), confirming the pH responsiveness of NPs at tumor-relevant pH. While the steady release of IL-12 at pH 7.4 is somewhat unexpected, probably due to the diffusion through the polymeric matrix, we expect that NPs will sufficiently accumulate into the tumor and release IL-12 rapidly within the acidic TME.

### IL-12-loaded pH-responsive NPs re-polarize mouse and human macrophages in vitro

**Fig. 2:**
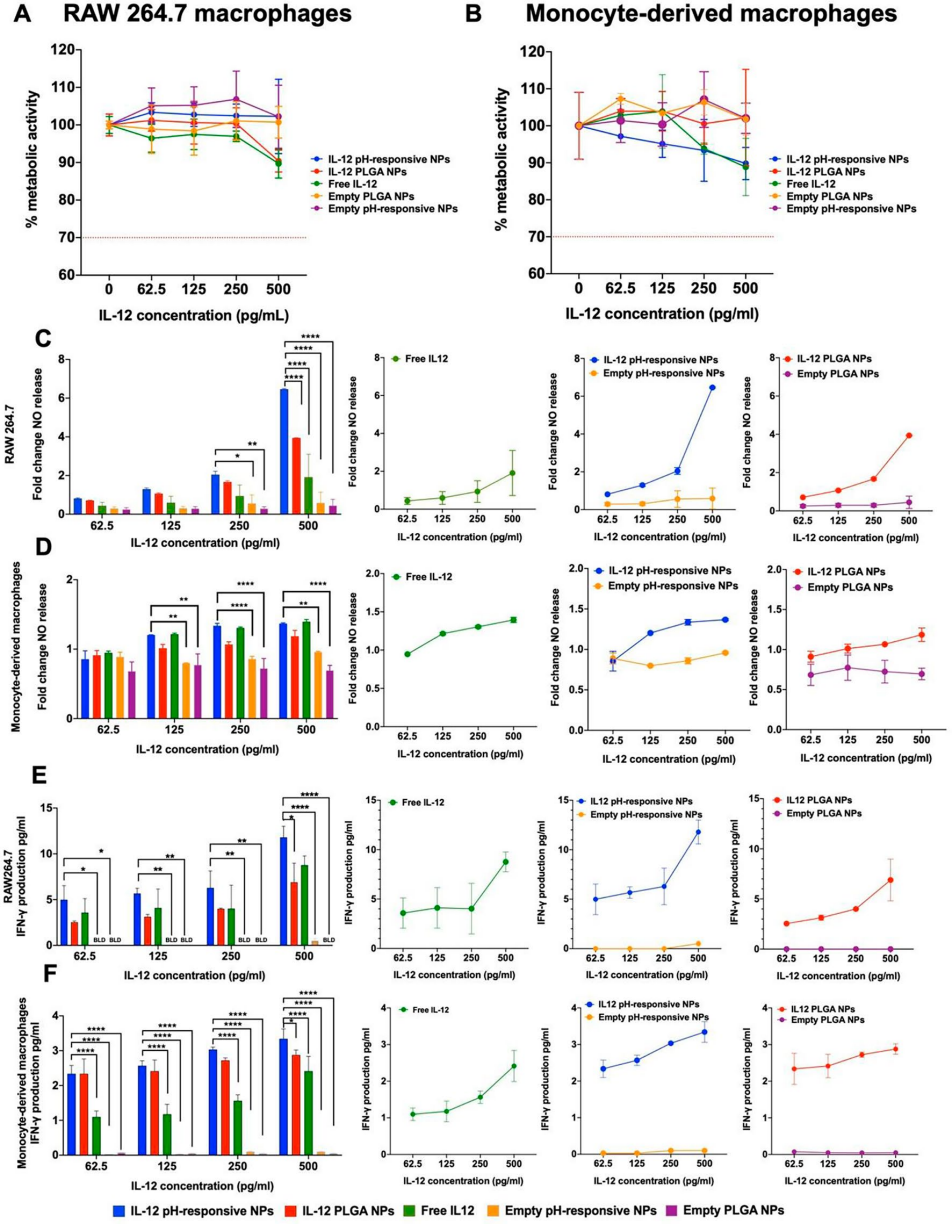
Evaluation of the impact of the IL-12 pH-responsive NPs in macrophages. **A** and **B**) Metabolic activity assessment in murine RAW264.7 macrophages (left panel) or human monocyte-derived macrophages (right panel) after 24 h of incubation with and IL-12-loaded pH-responsive NPs and the corresponding controls: IL-12 PLGA NPs (IL-12 pH non-responsive NPs), free IL-12, empty pH-responsive NPs and empty PLGA NPs (empty non-responsive NPs). **C** and **D**) Evaluation of the impact of different IL-12 pH-responsive NPs concentrations and corresponding controls in the NO release in 2D RAW264.7 macrophages or human monocyte-derived macrophages, respectively. **E** and **F**) Assessment of the IL-12 pH-responsive NPs and respective controls on the IFN-γ secretion levels in RAW264.7 macrophage cell line or human monocyte-derived macrophages, respectively. Statistical comparison of IL-12-loaded pH-responsive NPs with corresponding controls in each dose using one-way ANOVA, *p < 0.05, **p < 0.01, ***p < 0.001 or ****p < 0.0001. Other comparisons non-significant were *p* > 0.05

IL-12 can activate macrophages into pro-inflammatory phenotype by inducing the release of IFN-γ [41, 42]. We first examined the effect of the NPs on the metabolic activity of macrophages over 24 h. Different concentrations of IL-12 ranging from 62.5 to 500 pg/mL were incubated with both murine RAW264.7 macrophages and human monocyte-differentiated macrophages isolated from healthy donor buffy coats (Fig. 2A and B, respectively). As expected, there were no disparities in the metabolic activity of both mouse or human macrophages after incubation with IL-12-loaded pH-responsive NPs or respective controls (empty and IL-12 loaded pH-responsive NPs, empty and IL-12 loaded pH non-responsive PLGA NPs, and free IL-12).

Furthermore, no significant impact of the distinct NPs on the metabolic activity of pre-polarized macrophages (M1-/M2-like) was observed (Fig. S1). IL-12 induces the secretion of IFN-γ and thereby establish M1-like polarization by the activation of the expression of inducible NO synthase [43]. Therefore, the release of NO from macrophages was investigated (Fig. 2C and D). As presented in Fig. 2C and D, both RAW264.7 and human macrophages produced NO with increasing concentration of free IL-12. Interestingly, IL-12-loaded pH-responsive NPs showed a higher NO release compared to empty NPs and pH non-responsive NPs (Fig. 2C and D). Data showed that murine macrophages were more sensitive to IL-12 than human monocyte-derived macrophages which led to also lower differences in response of different NPs. These results demonstrate that both macrophage types reacted to higher levels of IL-12, acquiring an inflammatory M1-like phenotype, as corroborated by the fold change of NO release observed for pre-polarized M1-like macrophages (Fig. S2).

To further confirm the M1 polarization of macrophages, we examined the effect of the developed NPs on the secretion of IFN-γ from macrophages. As presented in Fig. 2E and F, IL-12 pH-responsive NPs effectively increased the levels of IFN-γ in both RAW264.7 and human primary macrophages across all tested concentrations, compared to the empty NP controls. Interestingly, significant differences were observed between pH-responsive NPs and non-responsive NPs with more IFN-γ production by the macrophages in response to the IL-12 pH-responsive NPs, reinforcing their specificity to respond to the slightly acidic pH (Fig. 2E). Despite the variability introduced by the isolation of macrophages from different blood donors, significant differences, particularly at the concentration of 500 pg/mL, were observed between IL-12 pH-responsive NPs and IL-12 non-responsive NPs (Fig. 2F). Of note, low response of free IL-12 might be due to its degradation while encapsulated IL-12 was stable for longer time, leading to enhanced overall response. Collectively, these findings support the ability of the developed NPs to drive macrophage polarization towards a M1-like anti-tumoral phenotype.

### Establishment and characterization of heterotypic CRC 3D (immuno)spheroids

**Fig. 3:**
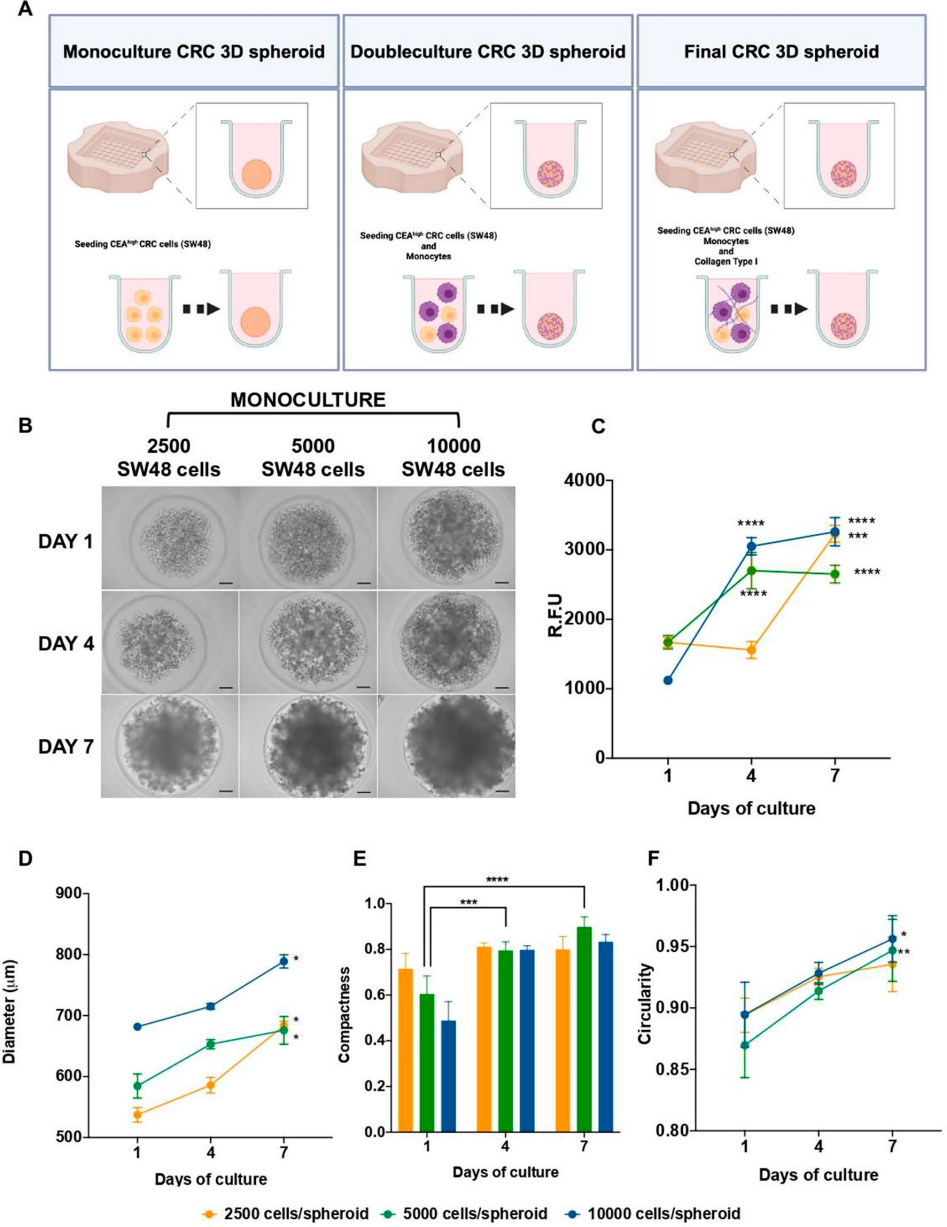
Establishment and characterization of SW48 monoculture 3D spheroids over a 7-day period. **A**) Diagram depicting the proposed final CRC spheroid 3D model containing SW48 CEA + CRC cells, macrophages differentiated in situ from peripheral blood monocytes, and collagen Type I, Generated using BioRender.com. **B**) Brightfield microscopy images illustrating the progression of 3D spheroids’ morphology over time, with three distinct initial cultured cell densities. The scale bar indicates 100 μm. **C**) Dynamics of the 3D spheroids’ metabolic activity; **D**) Changes in average diameter; **E**) Progression of compactness and **F**) Evaluation of circularity over time based on the initial seeding cell density. Values represent mean ± standard deviation (*n* ≥ 3). Statistical comparison performed between each cell concentration and days of culture

To evaluate the therapeutic potential of the developed NPs, a novel 3D CRC spheroid model was established to replicate the 3D structure of the CRC TME. This model incorporated SW48 CRC cells, human monocyte-derived macrophages, and collagen as an exogenous ECM component. CRC 3D spheroids were generated using microwell array technology by seeding cells into high-throughput 81-well agarose hydrogel micro-molds, promoting cell-cell aggregation and enabling large-scale, reproducible production of uniformly sized spheroids [30, 31]. Optimization involved seeding SW48 cells as a tumor core, followed by the addition of human monocyte-derived macrophages and collagen to better mimic in vivo conditions (Fig. 3A). SW48 cells were selected for their microsatellite instability (MSI)-high status and Consensus Molecular Subtype (CMS) 1 classification, reflecting CRC patients eligible for immunotherapy and making them an ideal model for testing immunomodulatory NPs [44, 45].

#### Establishment and optimization of the CRC mono-spheroid 3D model

To develop the SW48 CRC mono-culture 3D spheroids, cells were seeded at three different densities (2500, 5000, and 10,000 cells per spheroid) on agarose micro-molds, as recommended by the supplier and reported by previous studies [30, 46]. Afterwards, monoculture spheroids were qualitatively evaluated by brightfield microscopy revealing the formation of cell aggregates for all cell densities by day 1 (Fig. 3B). Over the subsequent 7 days, the integration of cell-cell interactions in a 3D cellular structure was observed in the higher cell densities, resulting in a more compact and circular structure (Fig. 3E and F) [47]. However, the characteristic was not achieved in the lower cell density. Additionally, monoculture spheroids were assessed in terms of metabolic activity along the 7 days of formation, presenting for the intermediate cell density (5000 cells/spheroid), a stable metabolic activity of approximately 2500 R.F.U. from day 4 to day 7 (Fig. 3C). Likewise, the average size was assessed, showing consistent values of around 650 μm from day 4 to 7 (Fig. 3D). In contrast, the densities of 2500 and 10,000 cells/spheroid exhibited increasing metabolic activity and average size, over the culture period, suggesting rapid growth at these densities, consistent with typical proliferation rates in 2D cellular cultures [48]. Therefore, the observed nearly constant levels of metabolic activity for a cell density of 5000 cells/spheroid were aligned with the typical steady growth rate of a 3D spheroid structure, being this selected as the most appropriate density to proceed with further studies [30]. In addition, to generate a more compact 3D structure with more cell-cell interaction and better mimic a more advanced tumor stage, 7 days of culturing was deemed the optimal condition for all further experiments.

Since previous studies reported the lack of α-catenin expression in SW48 cells, which is crucial for cell adhesion [49], 8 µg/mL of collagen was incorporated into the 3D CRC spheroids to promote a matrix-dependent cluster formation [50] (Fig. S3).

### Establishment of 3D heterotypic immuno-spheroid to study the effect on tumor-macrophages interaction

**Fig. 4:**
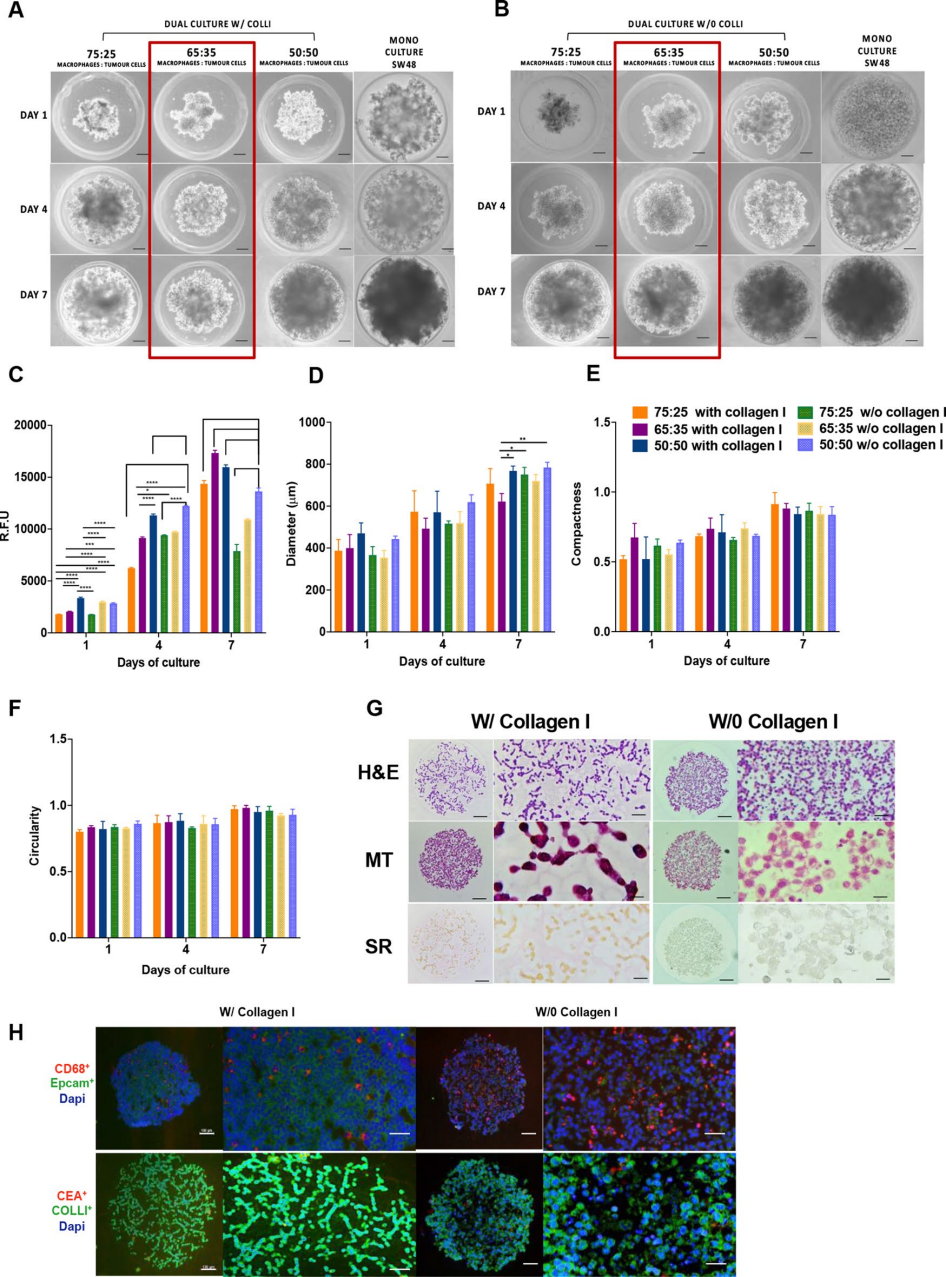
Temporal evolution and assessment of CRC 3D spheroids in co-culture with human macrophages, at various ratios, in the presence or absence of collagen. **A** and **B**) Brightfield microscopy depicting morphological changes in CRC 3D spheroids over time with different macrophage-to-tumor cell ratios with or without collagen (COLLI), respectively. Scale bar: 100 μm. **C**) Analysis of the impact of collagen in CRC 3D spheroids metabolic activity, **D**) diameter, **E**) compactness and **F**) circularity over a 7-day period compared to initial seeding cell density. Values are mean ± standard deviation (*n* ≥ 3). Statistical comparison performed between each cell concentration w/ or w/o collagen I and days of culture. **G**) H&E, MT and SR staining at 20× magnification (scale bar: 100 μm) and 40× magnification (scale bar: 50 μm) revealing the structure of the selected final ratio of CRC 3D spheroids on day 7. **H**) Immunofluorescence microscopy on day 7 displaying cellular organization (SW48-EpCAM in green, macrophages–CD68 in red) and co-localization of SW48-CEA expression (red) and ECM-collagen (green). Nuclei were counterstained with DAPI (blue). Scale bars: 100 μm

To mimic the tumor immune microenvironment, a dual-culture system of human monocytes-derived macrophages and SW48 tumor cells was established in three different ratios, namely 75:25, 65:35, and 50:50 (monocytes: tumor cells) in collagen. The compactness, circularity, diameter and metabolic activity of the spheroids were investigated until day 7 (Fig. 4). On day 1, as observed by brightfield microscopy, dual-culture 3D spheroids exhibited cell aggregates for all tested ratios, both with and without collagen (Fig. 4A and B). However, by day 7, there was an increase in cell compactness and the formation of a spherical 3D structure when cultured in collagen. For the 3D spheroids with collagen, in all ratios, the spheroids metabolic activity increased over time from day 1 to day 7, while spheroids without collagen showed no increase in the metabolic activity (Fig. 4C). These data show the effect of ECM, which supports the growth of tumor cells by providing cell-ECM interaction. Moreover, the interaction of the macrophages with the tumor cells and its penetration through the collagen network can also impact the metabolic activity of the spheroids [30]. Additionally, the cells present in the spheroids without collagen seemed to not be able to interact with each other to the same magnitude, avoiding their ability to proliferate and probably affecting the secretion of factors that impair immune cell viability [51]. Furthermore, the average size of the 3D CRC spheroids expanded in size over time across all tested cellular ratios (Fig. 4D). However, when comparing 3D spheroids with and without collagen, at the ratio 65:35 (monocytes: tumor cells) the ones with the presence of collagen exhibited a deceleration in the tumor diameter, exhibiting the smaller sizes of ~ 500 μm at day 4 to ~ 650 μm at day 7. Additionally, no biological relevant changes among the different ratios with collagen, at day 1, 4 and 7, in terms of compactness and circularity, were achieved (averaging 0.5-1, Fig. 4E and F). Accordingly, spheroids without collagen displayed slightly lower values of compactness and circularity (averaging 0.5–0.8, Fig. 4E and F). Considering the obtained results in terms of size and metabolic activity, the 65:35 ratio (monocytes: tumor cells) with collagen, was selected as the optimal condition for subsequent studies. To confirm the presence and co-localization of collagen, histological analyses including H&E, MT and SR staining, were conducted (Fig. 4G). The H&E histological analysis revealed a well-organized structure at day 7, with better compactness of the cells and spherical shape when collagen was present (Fig. 4G). In addition, the absence of collagen led to a more irregular 3D spheroid, exhibiting surface protuberances and lack of ECM support to sustain the 3D structure (Fig. 4G).

As expected, MT and SR staining allowed to successfully confirm the presence of collagen fibers as an exogenous ECM counterpart in the final dual-culture model (Fig. 4G). Moreover, SR, which typically stains collagen type I fibers in red color, validated the presence of ECM. Immunofluorescence studies were further performed to investigate the cellular location of SW48 cells (EPCAM^+^ and low CEA expression CEA^+^), macrophages (CD68^+^), and the ECM component (collagen^+^), as presented in Fig. 4H. The spatial organization of the spheroids demonstrated that macrophages are widely distributed throughout the 3D structure, rather than accumulating solely at the surface, with or without collagen. Moreover, macrophages were surrounded by SW48 tumor cells, which were associated with a certain degree of CEA expression, in accordance with our previous work [25]. The presence of collagen was found to be homogeneous throughout the spheroids, contributing to increased compactness of the structure (Fig. 4H). In 3D spheroids without collagen addition, collagen type I was still identified, although in lower amounts, suggesting minimum endogenous production of this ECM component by the cells of the spheroids. In summary, this 3D spheroids successfully recapitulated part of the immune-tumoral TME of CRC, serving as a promising tool for drug and NP testing.

#### Effect of IL-12 loaded NPs on cellular interactions and metabolic activity of the 3D CRC spheroids

The ECM is responsible for generating physical barriers and diffusion gradients in tissues, which might affect the NPs interactions and compromise their overall effectiveness [52]. To validate these findings in a more complex 3D model of CRC, the 3D CRC spheroids were incubated with the NPs to study their cellular uptake and effect on proliferation. Notably, the pH responsiveness of the NPs did not affect their cellular uptake, with no significant differences found between non-responsive and pH-responsive NPs (Fig. S4). The results obtained are consistent with our findings, demonstrating no dependence between pH responsiveness and cellular uptake. The ability of IL-12-loaded pH-responsive NPs to inhibit CRC 3D spheroids cell viability was assessed after a 24 h period of treatment with doses ranging from 62.5 pg/mL to 500 pg/mL (Fig. S5). The NPs did not impact the metabolic activity of the CRC 3D spheroids at any tested concentration, consistent with observations in the 2D assays (Fig. S5). Moreover, no significant differences were observed between the developed NPs and free IL-12. Overall, as previously attained in 2D studies, NPs were deemed safe for macrophages.

#### Effect of IL-12 loaded NPs on the macrophage re-polarization in 3D immuno-spheroids

**Fig. 5:**
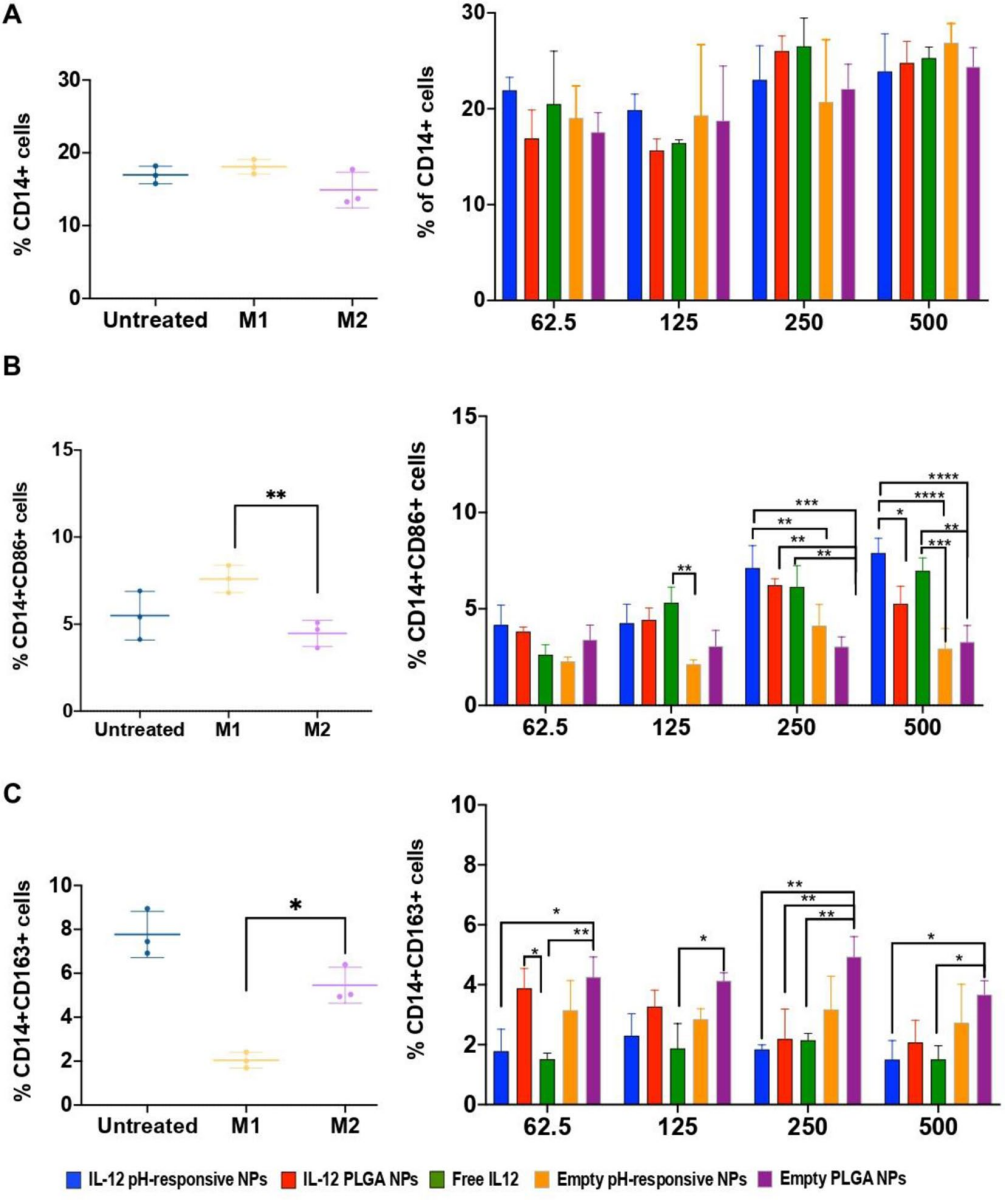
Evaluation of macrophage polarization profile in the CRC 3D spheroids before and post-treatment **A**) Analysis of the CD14 + macrophage population. **B**) Assessment of the expression of CD86 (M1-like marker) and **C**) CD163 (M2-like marker), presented as a percentage of expressing positive cells. The CRC 3D spheroids were incubated with various treatments, such as cell culture medium (untreated), unloaded PLG NPs and pH-responsive NPs, free IL-12, and IL12- PLGA NPs and IL-12 pH-responsive NPs for 24 h. Statistical comparison between IL-12-loaded pH-responsive NPs and the respective control groups was conducted within each dose range. Significance levels are denoted as *p < 0.05, **p < 0.01, ***p < 0.001 or **** *p* < 0.0001

To investigate the effect of IL-12 pH-responsive NPs on macrophage re-polarization within the CRC 3D immuno-spheroids, a range of concentrations of NPs, in terms of IL-12 were assessed for a 24 h period of treatment. The analysis of the CD14 + macrophage population in CRC 3D spheroids was focused on assessing the expression of CD163+ (anti-inflammatory M2 marker) and CD86+ (a pro-inflammatory M1 marker), as observed in Fig. 5. As presented in Fig. 5A, there were no significant variations in the expression of the CD14 + macrophage population across all experimental groups, maintaining a consistent percentage of monocytes/macrophages of around 19.3 ± 0.7%. However, a notable difference between the initially seeded cells of 65% monocytes to 19% macrophages was observed, which can be explained by the non-proliferative nature inherent to macrophages. Interestingly, the final ratio aligns with the in vivo context of CRC, characterized by ~ 18.21% TAMs [53].

With increasing concentrations, IL-12-loaded pH-responsive NPs showed an increasing expression levels of CD86 which were significantly higher than pH non-responsive NPs, at least at the highest concentration (Fig. 5B). These data suggest a successful release of bioactive IL-12 over time and its ability to re-polarize macrophages into anti-tumoral phenotype. Additionally, similar expression of CD86 was observed between the pre-polarized M1-like macrophages (LPS and IFN-γ stimulation) and macrophages treated with IL-12 non-responsive NPs and free IL-12 (Fig. 5B). However, no significant differences in CD86 expression were found in the CRC 3D spheroids treated with 250 pg/mL or 500 pg/mL IL-12 pH-responsive NPs *versus* the treatment with equivalent concentrations of free IL-12 (Fig. 5B). Regarding the pre-polarization of the CD14 + population towards the M2-like phenotype, a reduction in CD163 expression was observed for all conditions treated with IL-12 pH-responsive NPs and free IL-12 (Fig. 5C). Moreover, similar findings of CD163 expression were found for the CD14 + population pre-polarized towards the M2-like phenotype (IL-10 stimulation).

In summary, the developed NP strategy clearly demonstrated the specificity in inducing a pro-inflammatory M1-like profile in macrophages, characterized by higher CD86 and lower CD163 expression. Importantly, compared with the free IL-12, the bioactivity of the IL-12 released from the pH-sensitive NPs was kept.

#### Effect of IL-12 loaded pH-responsive NPs on the secretion of IFN-γ and IL-10 in 3D immuno-spheroids

**Fig. 6:**
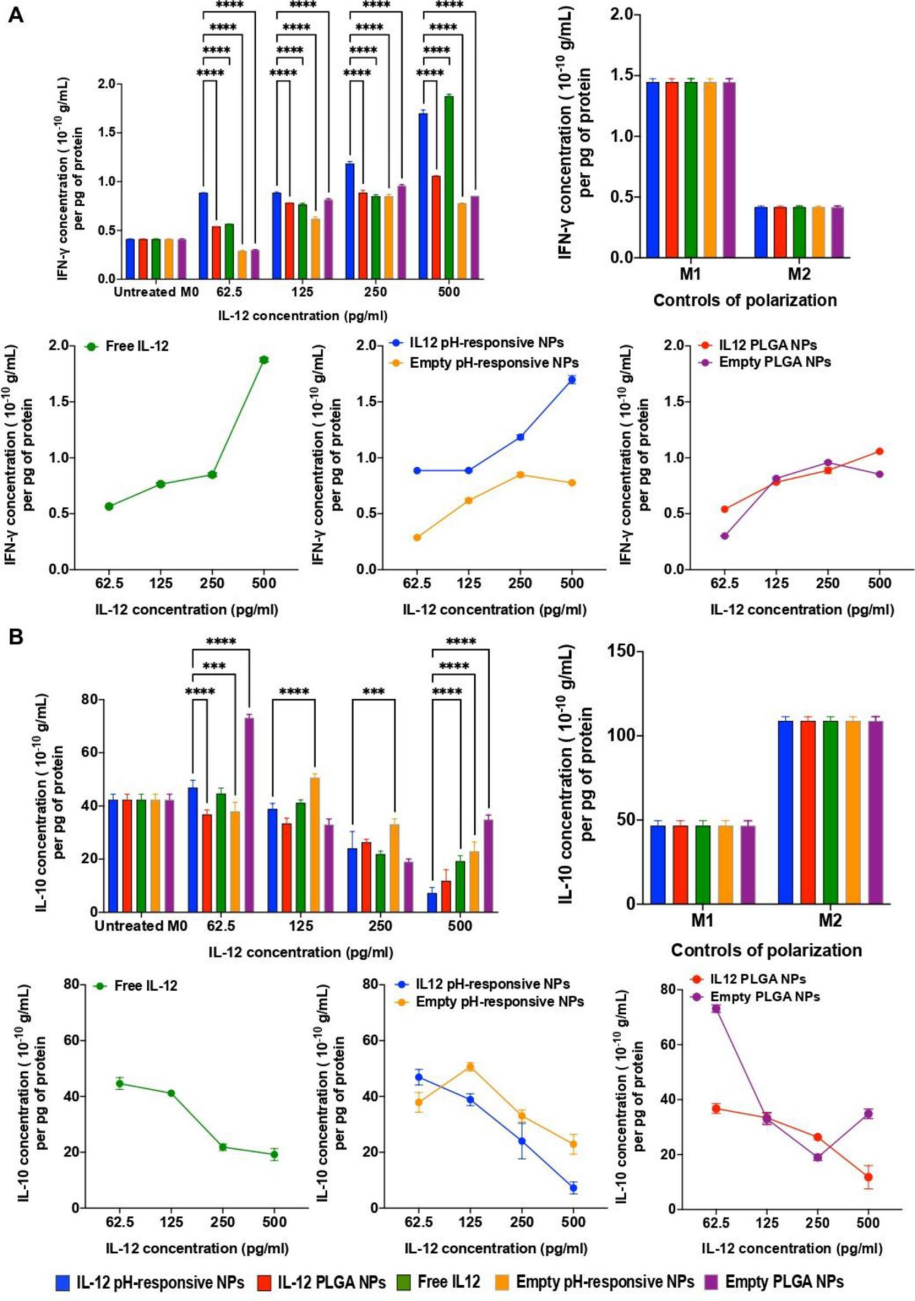
Evaluation of the potential of the developed strategy to induce pro-inflammatory and anti-inflammatory cytokine production in a CRC 3D spheroid after a 24 h treatment, comparing with the controls. **A**) Assessment of the potential of the developed NPs to induce IFN-γ production (left), assessment of the IFN-γ production in the controls of M1 or M2-like stimulated macrophages (right) and comparison between conditions (bellow).**B**) Analysis of the secretion of the anti-inflammatory cytokine IL-10 induced by the treatments (left) Assessment of the IL-10 production in the controls of M1 or M2-like stimulated macrophages(right) and comparison between conditions (bellow). Statistical comparisons were performed between the different testing groups, denoted as *p < 0.05, **p < 0.01, ***p < 0.001, or *p* < 0.0001

To validate the hypothesis that the developed NPs can induce a macrophagic M1-like phenotype, levels of cytokines associated with this polarization, such as IFN-γ, were assessed (Fig. 6A). Moreover, to validate the pro-inflammatory polarization of macrophages in CRC 3D immuno-spheroids, the evaluation of anti-inflammatory cytokines such as IL-10 was conducted, as its secretion is associated with M2-like macrophages and tends to decrease during the opposite M1-like polarization (Fig. 6B) [54]. Of note, the intrinsic ability of CRC 3D spheroids to independently secrete signaling mediators was confirmed through untreated controls of M1- or M2-like pre-polarized macrophages (Fig. 6A and B right, respectively). Changes in the secretion of IFN-γ and IL-10 were evaluated after a 24 h treatment. As depicted in Fig. 6A, for all tested concentrations, IL-12-loaded pH-responsive NPs were able to induce IFN-γ production by macrophages, with significant differences when comparing IL-12 pH-responsive NPs and the respective controls. Although there was no statistically significant difference in the levels of IFN-γ secreted between conditions treated with IL-12 pH-responsive NPs and free IL-12, the conditions treated with IL-12 pH-responsive NPs exhibited higher IFN-γ secretion levels, highlighting a promising trend (Fig. 6A). Moreover, although empty NPs presented no biological relevance, they showed similar trends to free IL-12 at lower concentrations, an unexpected finding, possibly linked to minimal cellular uptake or direct NP - membrane interactions that, by themselves, could activate signaling cascades through surface receptors and trigger a mild pro-inflammatory response.

Furthermore, the pro-inflammatory potential of the developed NP strategy was evaluated by assessing the anti-inflammatory IL-10 levels, as shown in Fig. 6B. A significant reduction was found between conditions treated with IL-12 pH-responsive NPs and the controls, with lower IL-10 levels observed, particularly for the higher concentrations (250 and 500 pg/mL). Moreover, significant differences were observed when comparing IL-12 pH-responsive NPs with IL-12 non-responsive NPs and the empty NP control (Fig. 6B). In summary, the developed IL-12 pH-responsive NPs successfully induced IFN-γ production, suggesting the re-polarization of macrophages towards a M1-like phenotype and inhibition of M2-like phenotype, shown with the reduced IL-10 levels.

Altogether, these results provided evidence that the developed IL-12 pH-responsive NPs could enhance, under low pH conditions, pH ~ 6.5, the release and bioactivity of IL-12. Therefore, the release of IL-12 was able to drive macrophages into a more pro-inflammatory phenotype, with increased expression of CD86 and secretion of IFN-γ, while reducing the expression of CD163 and secretion of IL-10.

## Conclusions

In this study, we have successfully developed a novel pH-responsive nanoplatform loaded with IL-12 and demonstrated its bioactivity in biorelevant 2D and 3D culture models, mimicking the CRC TME features. The manufacturing of the pH-responsive NPs involved, a priori, the chemical modification of the PLGA core polymer with the pH-responsive DMMA monomer, whose hydrolysis under weakly acidic pH (such as in the CRC TME) exposes its amino groups. IL-12 was effectively encapsulated into the pH-responsive NPs, and its pH-triggered release was confirmed under slightly acidic pH conditions, comparable with the CRC TME. IL-12 pH-responsive NPs increased NO release levels by 2-3-fold, as well as IFN-γ secretion, in both 2D mouse and human primary macrophages differentiated from monocytes isolated from healthy donor buffy coats. These results suggested that IL-12 pH-responsive NPs can trigger the stimulus for M1-like macrophage polarization. Additionally, a novel CRC 3D spheroid model was developed, composed by SW48 cancer cells, macrophages differentiated in situ from peripheral monocytes, and an exogenous ECM-like non-cellular constituent, collagen. The CRC 3D spheroids demonstrated the ability to mimic cellular and structural features of the tumor, such as cell spatial organization, presence of ECM, and a pro-tumor and anti-inflammatory M2-like macrophage phenotype. Moreover, the effect of the IL-12 pH-responsive NPs was then investigated in the CRC 3D immuno-spheroid model, in which the pH-responsive NPs reduced the expression of the anti-inflammatory CD163 macrophage marker, while increasing the levels of the pro-inflammatory CD86 macrophage marker, hence confirming the ability to reeducate the tumor immune microenvironment. Accordingly, IL-12 pH-responsive NPs led to a reduction in the secretion of a key anti-inflammatory cytokine, IL-10, and an increase of the IFN-γ pro-inflammatory cytokine.

Overall, the IL-12 pH-responsive NPs allowed the faster release of IL-12 at the TME-relevant acidic pH (~ 6.5) than the physiological pH, while this aspect needs improvement in view of higher stability at pH 7.4 while achieving a sharpened release at pH 6.5. The IL-12 pH-responsive NPs were deemed safe for macrophages and showed pro-inflammatory ability to induce IFN-γ production. Moreover, the established CRC 3D immuno-spheroid model presents a new in vitro tool to better recapitulate the biological architecture and composition of the native tumor, making it a promising technology for the testing of newly developed (nano-)drugs with better in vivo predictability.

## Electronic supplementary material

Below is the link to the electronic supplementary material.

Supplementary Material 1

## Data Availability

The collected and analyzed datasets during this study are available from the corresponding author on reasonable request.
