# Supplementary material for: Immunostimulatory effects of IL-12 targeted pH-responsive nanoparticles in macrophage-enriched 3D immuno-spheroids in vitro model
Source: Drug Deliv Transl Res. 2025 Jun 16;15(12):4775–94. doi: 10.1007/s13346-025-01896-8 (PMC12619765; doi:10.1007/s13346-025-01896-8)
Supplement: Supplementary file 1 — Supplementary Material 1 [file 13346_2025_1896_MOESM1_ESM.docx]

**Supplementary Information**

Figure S1: Evaluation of the impact of the developed NPs and respective controls on the metabolic activity. of macrophages polarized towards M1 or M2-like phenotypes

Figure S2: Evaluation of the fold change NO release on the controls of macrophages polarized into an M1 or M2-like status.

Figure S3: Evaluation of the optimal concentration of collagen I within three different cell ratios in monocultures CRC 3D spheroids. Diameter size assessment was conducted over the course of 7 days of culture.

Figure S4: Flow cytometry analysis of the percentage of IL-12 pH-responsive NPs and the respective controls internalized by the CRC 3D spheroids.

*Figure S5: Evaluation of the impact of IL-12 pH-responsive NPs and the corresponding controls on the metabolic activity of CRC 3D spheroids.*
